# Paradoxical Glucose-Sensitizing yet Proinflammatory Effects of Acute ASP Administration in Mice

**DOI:** 10.1155/2013/713284

**Published:** 2013-05-12

**Authors:** Alexandre Fisette, Pegah Poursharifi, Katerina Oikonomopoulou, Mercedes N. Munkonda, Marc Lapointe, Katherine Cianflone

**Affiliations:** ^1^Centre de Recherche de l'Institut Universitaire de Cardiologie et de Pneumologie de Québec, Université Laval, Y4332, 2725 Chemin Ste-Foy, Québec, QC, Canada G1V 4G5; ^2^Department of Pathology & Laboratory Medicine, School of Medicine, University of Pennsylvania, PA 19104-6100, USA

## Abstract

Acylation stimulating protein (ASP) is an adipokine derived from the immune complement system, which stimulates fat storage and is typically increased in obesity, type 2 diabetes, and cardiovascular disease. Using a diet-induced obesity (DIO) mouse model, the acute effects of ASP on energy metabolism and inflammatory processes *in vivo* were evaluated. We hypothesized that ASP would specifically exert proinflammatory effects. C57Bl/6 wild-type mice were put on a high-fat-high-sucrose diet for 12 weeks. Mice were then subjected to both glucose and insulin tolerance tests, each manipulation being preceded by recombinant ASP or vehicle (control) bolus injection. ASP supplementation increased whole-body glucose excursion, and this was accomplished with reduced concomitant insulin levels. However, ASP did not directly alter insulin sensitivity. ASP supplementation induced a proinflammatory phenotype, with higher levels of cytokines including IL-6 and TNF-*α* in plasma and in adipose tissue, liver, and skeletal muscle mRNA. Additionally, ASP increased M1 macrophage content of these tissues. ASP exerted a direct concentration-dependent role in the migration and M1 activation of cultured macrophages. Altogether, the *in vivo* and *in vitro* experiments demonstrate that ASP plays a role in both energy metabolism and inflammation, with paradoxical whole-body glucose-sensitizing yet proinflammatory effects.

## 1. Introduction

The past decades of obesity research have seen a change of paradigm. Adipose tissue was once labeled as a passive storage organ, while obesity comorbidities were predominantly linked with energy metabolism disorders. Currently, it is widely recognized that adipose tissue also plays a major endocrine role and that progressive metabolic diseases such as type 2 diabetes and atherosclerosis manifest a strong immune component. Moreover, molecules secreted by the expanding adipose tissue modulate systemic inflammation and contribute heavily to the pathology of the aforementioned diseases [1].

 Acylation stimulating protein (ASP), identical to C3adesArg, is a protein generated through the alternative complement pathway of the immune system [2]. C3a/C3adesArg is rapidly produced during acute immunological responses such as bacterial infection, which triggers the classical or lectin pathways of the complement system. However, in the absence of acute immune response, spontaneous activation of the alternative pathway of complement, mediated through adipsin, factor B, and complement C3 convertase, leads to the generation of C3a. C3a is rapidly desarginated by carboxypeptidase B or N to generate C3adesArg, termed ASP based on its specific metabolic effects. ASP acts through its only known receptor, C5L2. This 7-transmembrane receptor is widely expressed, with expression in tissues such as liver, muscle, adipose tissue, immune cells, and brain, and C5L2 shares significant homology with other complement receptors such as C3a receptor and C5a receptor [3].

*In vitro*, the functional role of ASP/C3adesArg has been extensively studied on adipocytes. Its primary effect is the promotion of fat storage, accomplished by increasing diacylglycerol acyltransferase activity [4] and GLUT4 translocation [5, 6], which drive fatty acid and glucose uptake into cells and provide the substrates for triglyceride synthesis. These effects have been shown to be independent and additive to those of insulin [7]. In adipocytes, ASP action is mediated through activation of PI3kinase, phospholipases C and A2, Akt phosphorylation, and protein kinase C [8]. 

The presence of C5L2 is required for ASP response in adipocytes [9–12], but in other cell types, the interaction of ASP with C5L2 remains controversial. However, C5a is also a ligand for both C5L2 and C5aR [13–15]. While C3a binds C3a receptor (C3aR), initiating a potent proinflammatory signaling cascade, C3adesArg does not bind C3aR, and it has canonically been considered as an inert fragment, indicative of complement system activation, and subsequent proteolytic cleavage of C3a [16]. However, C3adesArg has also been shown to be implicated in several immune processes, with less potency than C3a [17–20]. For example, C3adesArg retains indirect chemotactic properties on hematopoietic cells [19] and can also induce cytokine secretion by cultured macrophages and modulate liver regeneration [20]. 

ASP has been shown to possess a metabolic role in both humans and rodents. Acute postprandial production of ASP within the adipose tissue bed has been demonstrated in human studies, and this acute production directly correlates with dietary fatty acid uptake into adipose tissues [21, 22]. By contrast, ASP levels are chronically increased in obesity, type 2 diabetes, and cardiovascular diseases in humans [23], and higher circulating levels of fasting ASP in humans are associated with a delayed clearance of postprandial triglyceride [24]. This suggests that acute ASP responses may be very different from the metabolic profile associated with chronically elevated ASP, and the exact consequences of acute versus chronic ASP in these diseases remain unclear. 

The aim of the present study was to assess the effects of acute ASP administration on *in vivo* glucose metabolism and rapid inflammatory responses in a diet-induced obesity (DIO) mouse model. We hypothesized that ASP could modulate both adipose inflammation and alter systemic glucose disposal.

## 2. Materials and Methods

### 2.1. Animals

Male C57Bl/6 wild-type mice were obtained through breeding in our internal colony. All mice were individually housed in a sterile barrier facility with a 12 h light 12 h dark cycles. At 8 weeks, mice were placed on high-fat-high-sucrose (HF/HS) diet (58% kcal fat D12331; Research Diets Inc., New Brunswick, NJ, USA) for 12 weeks. All mice were weighed and randomly separated into two groups of equal body weight at week 11. Throughout the study, treated mice received a total of three ASP doses (high physiological) intraperitoneally (1.5 *μ*g/g body weight), and control mice received three PBS (vehicle) injections at the following times: (i) before glucose tolerance test (GTT) early week 11, (ii) ITT late week 11, and (iii) GTT sacrifice week 12, with a minimum of 5 days recovery between each injection. Blood was collected through cardiac puncture, and tissues were harvested and immediately frozen in liquid nitrogen. Tissues were subsequently transferred to −80°C and stored for further analysis. All protocols were approved and were conducted in accordance with the Canadian Council of Animal Care (CCAC) guidelines and approved by the Laval University Animal Care Committee.

### 2.2. Recombinant ASP

Recombinant human ASP was prepared as previously described, using a Ni^2+^-Sepharose column for initial purification via binding to the 6XHis-tag sequence at the amino-terminal, followed by HPLC purification as described in detail elsewhere [25]. No denaturing agents were used at any step in the purification to avoid ASP inactivation. Recombinant ASP purity was assessed with mass spectrometry and was endotoxin-free as evidenced by the Limulus amebocyte lysate (LAL) endotoxin assay (Lonza, Walkersville, MD). 

### 2.3. Plasma Analysis

Blood was collected through cardiac puncture. Plasma triglycerides, non-esterified fatty acids (NEFA), and glucose were measured using colorimetric enzymatic kits as follows: plasma triglyceride (Roche Diagnostics, Richmond, VA, USA), NEFA (Wako Chemicals, Richmond, VA, USA), and glucose (Sigma, Saint Louis, MO, USA). IL-6, IL-10, G-CSF, GM-CSF, KC, MCP-1, MIP-1*α*, TNF-*α*, insulin, leptin, and PAI-1 were measured using suspension bead array immunoassay kits following the manufacturer's specifications (Bio-Plex Pro Mouse Cytokine Assay 23-plex and Bio-Plex Pro Mouse Diabetes Assay 8-plex, Biorad, Mississauga, ON, Canada) on a Bio-Plex series 100 instrument (BioRad, Mississauga, ON, Canada), and information on intra- and interassay CVs IS provided by the manufacturer and is freely available.

### 2.4. Glucose and Insulin Tolerance Tests

A glucose tolerance test (GTT) was performed on mice (at week 11 of their diet) after an overnight fast. Following an intraperitoneal glucose injection (2 mg/g body weight) supplemented with/without ASP (1.5 *μ*g/g body weight), blood samples were taken at 0, 15, 30, 60, and 90 min. Mice were allowed 5 days to recover. An insulin tolerance test (ITT) was then performed following a 4-hour fast. Blood samples were taken at 0, 15, 30, 60, and 90 min after an intraperitoneal insulin injection (1 mU/g of body weight) supplemented with/without ASP (1.5 *μ*g/g body weight) as indicated. Glucose was measured using a colorimetric enzymatic kit (Sigma, Saint Louis, MO, USA), while insulin was measured using a RIA kit (Linco, St. Charles, MO, USA), and information on intra- and interassay CVs is provided by the manufacturer and is freely available.

### 2.5. Tissue Glucose Uptake

At the end of the 12-week diet protocol, overnight-fasted mice received an intraperitoneal injection of glucose (2 mg/g body weight) supplemented with radioactive deoxyglucose (0.5 *μ*Ci (^3^H) deoxyglucose/g of body weight) and ASP (1.5 *μ*g/g body weight) and were euthanized exactly 2 hours later. Radioactive glucose uptake in visceral adipose tissue, muscle, and liver was evaluated using (^3^H) deoxyglucose tracer uptake in tissue homogenates. Raw dpm results were corrected for total protein content (muscle and liver) or weight (adipose tissue) and are expressed as relative deoxyglucose uptake.

### 2.6. Tissue Lipid

Adipose tissue, liver and muscle triglycerides, and NEFA were extracted from tissues using heptane : isopropanol (3 : 2). The organic extract was transferred, while the remaining tissue (liver and muscle) was air dried, dissolved in 0.3 N NaOH, and assessed for protein content using the Bradford method (Bio-Rad, Mississauga, ON, Canada). Organic extracts were lyophilized, and lipids were redissolved in 10% Triton X-100 aqueous solution. Triglycerides and NEFA were measured using commercial colorimetric kits as described above. Results are expressed as *μ*moles of triglycerides or NEFA per gram (g) of protein.

### 2.7. Real-Time Quantitative PCR

Tissue or cell mRNA was extracted, purified, and reverse transcribed into cDNA using RNeasy Mini Kits or RNeasy Lipid Tissue Mini Kits and QuantiTect Reverse Transcription Kits (Qiagen, Gaithersburg, MD, USA). mRNA for *F4-80*, *CD11c*, *CD163*, *IL-6*, *KC*, *MCP-1*, *MIP-1*α**, *PAI-1*, and *TNF-*α** was quantified using custom primers (Table 1). Relative gene expression was calculated and corrected using *GAPDH* (QuantiTect Primer Assay, Qiagen, Gaithersburg, MD, USA) as housekeeping gene. All procedures followed the manufacturer's instructions and minimum information for publication of quantitative real-time PCR experiments (MIQE) guidelines [26].

### 2.8. Macrophage Migration

Macrophage migration was assessed using QCM Chemotaxis 5 *μ*M 24-well cell migration assay (Millipore, Billerica, MA, USA) following the manufacturer's instructions. Briefly, confluent RAW 264.7 cells were harvested and resuspended in serum-free media, and 1 × 10^6^ cells were added to each insert. Adipocyte-conditioned media was obtained by incubating 3T3-L1 differentiated adipocytes overnight in serum-free DMEM/F12. 3T3-L1-conditioned media or serum-free medium control was then added to the lower chambers, with or without ASP (50 nM or 200 nM). Cells were incubated for 20 hours at 37°C in a CO_2_ incubator. Macrophages that migrated through the membrane were quantified following the manufacturer's instructions.

### 2.9. Macrophage Polarization

RAW 264.7 cells were seeded in 25 cm² flasks (1 × 10^6^ cells per flask) in DMEM/F12 with 10% FBS medium. Cells were incubated for 24 h to permit attachment and cultured to reach 80% confluence. Flasks were then subjected to a combination of a polarization treatment with/without a fixed ASP concentration. Polarization treatments were as follows: *Control treatment* consisted of serum-free media; *M1-polarization treatment* consisted of 100 ng/mL LPS and 100 ng/mL of IFN-*γ*; *M2-polarization treatment* consisted of 10 ng/mL of IL-4 and 10 ng/mL of IL-13. Concentrations of ASP used in conjunction with each treatment were 0 nM (baseline), 50 nM (low physiological), and 200 nM (high physiological). Cells were incubated 6 hours at 37°C in a CO_2_ incubator. After incubation, cells were immediately lysed, and mRNA was extracted.

### 2.10. Statistical Analysis

Results are expressed as mean ± SEM. Groups were compared using the appropriate statistical test, either one-way ANOVA with linear trend post hoc test, two-way ANOVA with Student-Newman-Keuls post hoc test, or *t*-test using Prism 5.0 software (GraphPad, CA, USA). Statistical significance was set as *P* < 0.05, where *P < 0.05, **P < 0.01, and ***P < 0.001 and where 0.05 < *P* < 0.1 was considered a tendency.

## 3. Results

### 3.1. ASP Injections Did not Alter Body Weight or Food Intake

Mice were injected with high-physiological doses of ASP at the indicated time periods, as described in the methods. No change in body weight or food intake between ASP-treated animals and controls was recorded during the 12-week treatment period. 

### 3.2. ASP Alters Substrate Partitioning

**Figure 1:**
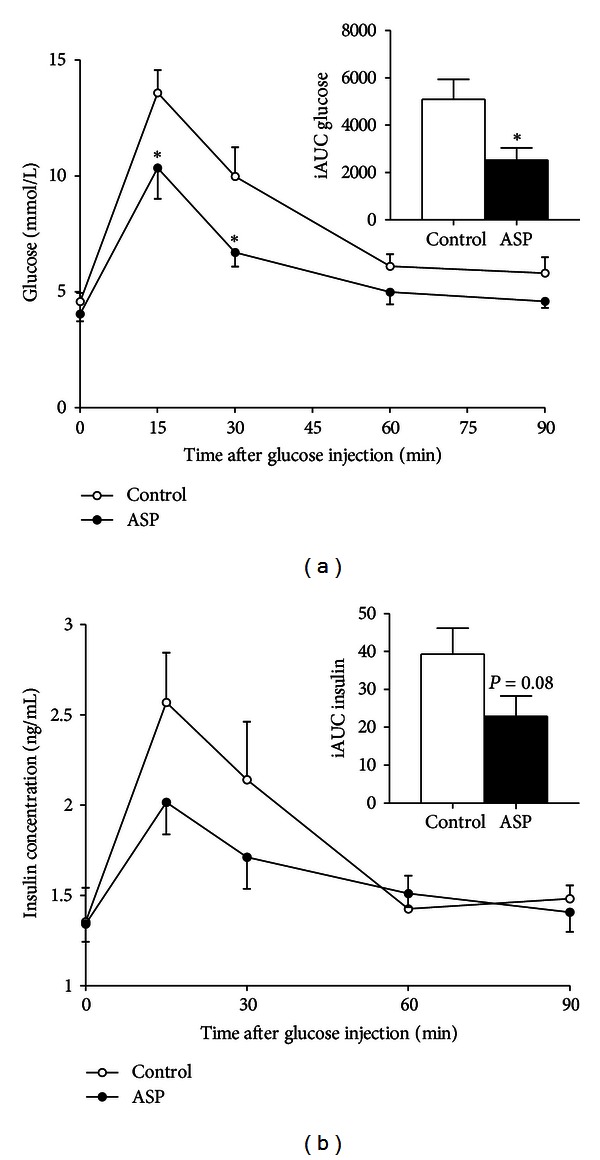
Glucose excursion is altered by ASP. Glucose with/without ASP was administered, and plasma glucose and insulin were measured over 90 minutes. Glucose levels are shown in (a) and concomitant insulin levels in (b). Incremental area under the curve for each graph is shown in the insert. Data are presented for glucose (mmol/L, (a)) and insulin (ng/mL, (b)). Results are expressed as mean ± SEM where differences versus controls (vehicle) are expressed as *P < 0.05, with *n* = 7-8 per group.

An intraperitoneal glucose tolerance test (GTT) with/without ASP was performed. The recombinant ASP dose was chosen to represent a high-physiological concentration of endogenous ASP, such as seen in severely obese individuals [22]. Glucose and insulin levels were monitored throughout a 90-minute timeframe after the injection (Figure 1). A more efficient glucose clearance was achieved with acute ASP supplementation (Figure 1(a), *P* < 0.05). Concomitant insulin levels showed a statistical tendency to be lower in the ASP-treated mice (Figure 1(b), *P* = 0.08). In order to assess if ASP acutely influenced insulin sensitivity, an intraperitoneal insulin tolerance test (ITT) was performed in the same animals, supplemented or not with ASP. No difference in insulin-mediated glucose response was detected in mice treated acutely with/without ASP (data not shown). 

**Figure 2:**
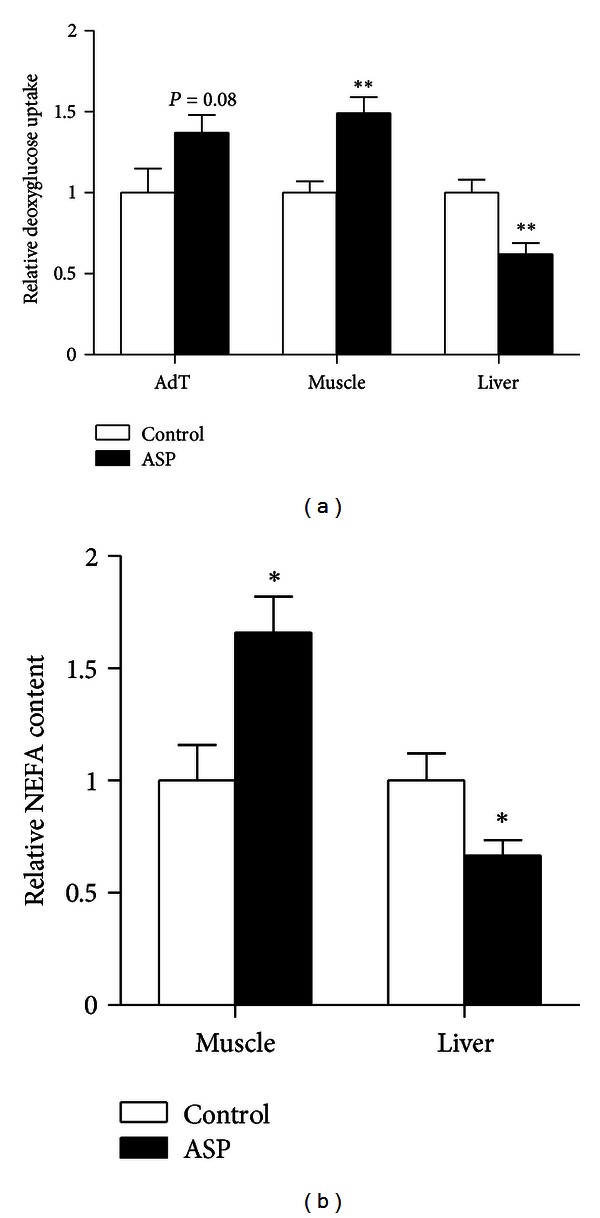
ASP affects substrate partitioning. Relative ^3^H-deoxyglucose uptake in adipose tissue (AdT), liver and skeletal muscle is shown in (a). Relative nonesterified fatty acid content of liver, and skeletal muscle is shown in (b). Results are expressed as relative absorption or content compared with controls (vehicle) which are set as 1.0. Results are expressed as mean ± SEM where differences versus controls (vehicle) are expressed as *P < 0.05 and **P < 0.01, with *n* = 7-8 per group.

A GTT with (^3^H) deoxyglucose supplemented with/without ASP was used to evaluate ASP effects on glucose uptake in specific tissues. Acute ASP treatment altered glucose uptake in a tissue-specific fashion; tissues relying mostly on active transport showed increased or tended to increase glucose uptake (adipose tissue, *P* = 0.08, and skeletal muscle, *P* < 0.01), while the liver, relying mainly on passive transport, exhibited lower glucose uptake (*P* < 0.01), as shown in Figure 2(a).

Acute ASP treatment did not alter muscle or liver triglyceride (data not shown). However, cellular NEFA content was increased in skeletal muscle (*P* < 0.05) and reduced in liver (*P* < 0.05), as seen in Figure 2(b). Circulating NEFA was decreased as well (*P* < 0.05) (Table 2).

### 3.3. ASP Increased Plasma Cytokine Concentrations and Altered Liver and Adipose Tissue Cytokine Expression

**Figure 3:**
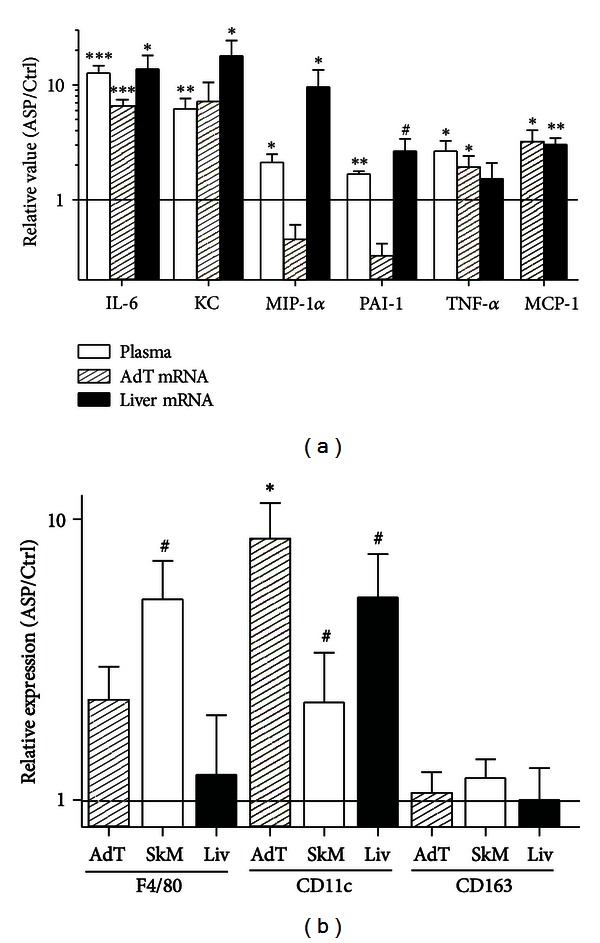
ASP induces proinflammatory effects *in vivo*. Relative plasma levels, adipose tissue, and liver mRNA content of several cytokines are shown in (a), for mice that were injected with ASP as compared to controls (PBS as vehicle). For the same animals, relative mRNA levels of total macrophages marker (F4/80), M1 macrophages marker (CD11c), and M2 macrophage marker (CD163) within adipose tissue, liver, and muscle are shown in (b). Results are expressed as relative concentration or expression compared with controls (vehicle) which are set as 1.0. Results are expressed as mean ± SEM where differences versus controls are expressed as ^#^ *P* = 0.08, *P < 0.05, **P < 0.01, and ***P < 0.001, with *n* = 7-8 per group.

The ASP bolus also induced acute changes in adipokine and cytokine profiles. Plasma leptin was reduced, while several inflammation-related cytokines including IL-6, IL-10, PAI-1, TNF-*α*, KC, and MIP-1*α* were significantly increased (Figure 3(a), Table 2). mRNA levels for these same cytokines were evaluated in liver and adipose tissue, with increased expression of IL-6, KC, TNF-*α*, and MCP-1 in both tissues and increased MIP-1*α* and PAI-1 in liver (Figure 3(a)). A global inflammatory phenotype is clearly induced by acute ASP injection in obese mice, mediated by both adipose tissue and liver changes.

### 3.4. Macrophage Infiltration and Polarization Is Influenced by ASP

Macrophage infiltration and polarization were evaluated in adipose tissue, skeletal muscle, and liver. As shown in Figure 3(b), macrophage marker F4/80 was not significantly altered by ASP treatment, although it tended to increase in the skeletal muscle (*P* = 0.07). No change was seen in anti-inflammatory M2 polarization marker (CD163). However, in all tissues, proinflammatory M1 polarization marker was significantly higher or tended to increase (adipose tissue, *P* < 0.05, skeletal muscle, *P* = 0.07, liver, *P* = 0.10), suggesting a preferential activation of infiltrating macrophages to an M1 phenotype with acute ASP treatment.

**Figure 4:**
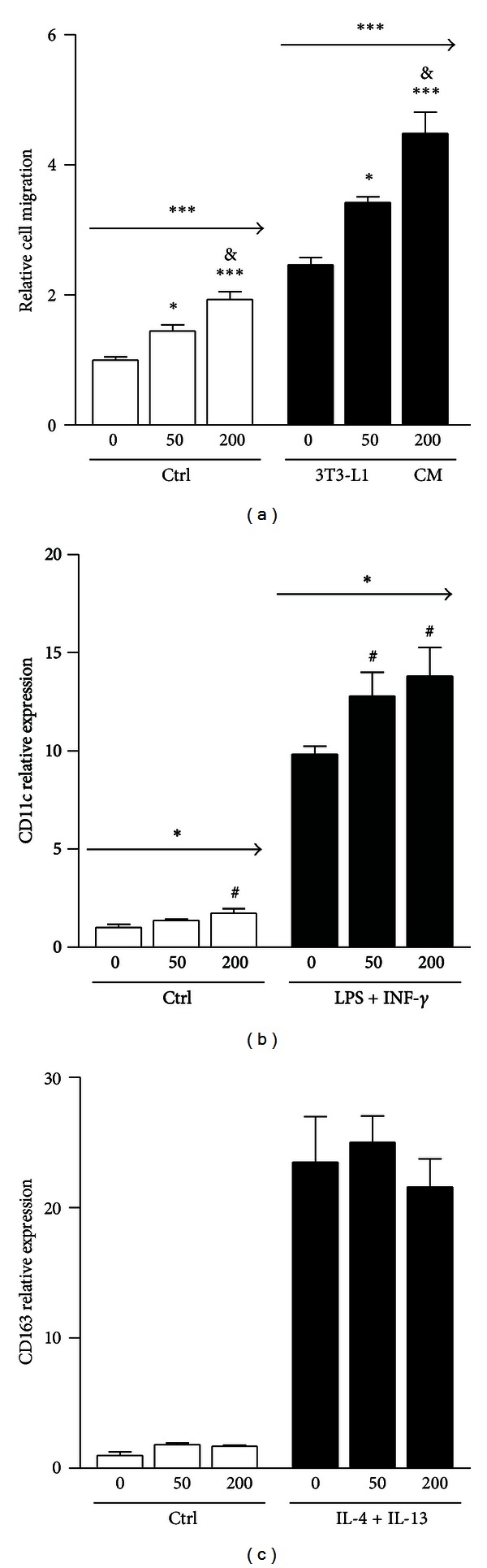
ASP induces proinflammatory effects *in vitro*. In (a), *in vitro* chemoattractive effects of three concentrations of ASP combined or not with adipocyte conditioned media on macrophages are shown. ASP effects on macrophage polarization to M1 phenotype are shown using M1 macrophage marker CD11c in (b) while ASP effects on M2 polarization are shown using M2 macrophage marker CD163 in (c). Results are expressed as relative expression or migration compared with Controls. Results are expressed as mean ± SEM where differences versus Control are expressed as ^#^ *P* = 0.08, *P < 0.05, **P < 0.01, ***P < 0.001, with  *n* = 4. & are used in (a) to indicate a *P* < 0.05 significant difference in between 200 nM and 50 nM ASP concentration effect. Arrows are used to indicate a significant linear trend by ANOVA analysis where *P < 0.05 and ***P < 0.001.

In order to further investigate the direct role of ASP in the physiological changes observed in mice, *in vitro* effects of ASP on macrophage migration and polarization in RAW 264.7 macrophages were evaluated. ASP showed dose-dependent chemoattractant migration properties when incubated alone with macrophages (Figure 4(a)), and these effects were further additive to those of 3T3-L1 adipocyte-conditioned media. ASP by itself had very limited capacity to induce macrophage M1 or M2 phenotype polarization (Figures 4(b) and 4(c)). However, RAW 264.7 macrophages treated with INF-*γ* and LPS to stimulate M1 polarization, as assessed by CD11c mRNA expression (Figure 4(b)), showed further M1 polarization with ASP treatment, in a dose-dependent fashion (Figure 4(b)). By contrast, while treatment with IL-4 and IL-13 increased M2 polarization, as assessed through CD163 mRNA expression (Figure 4(c)), ASP did not further enhance this effect.

## 4. Discussion

In the present study, the inflammatory role of acute high-physiological ASP administration in a context of established obesity was evaluated. Previous studies have focused either on the metabolic role of ASP in adipocytes or the immune role of ASP/C3adesArg in immune cells. This study examines a broader, more integrative physiological role of ASP. This study clearly demonstrated that ASP/C3adesArg acutely enhances glucose-insulin clearance mechanisms in spite of its acute paradoxical *in vivo* and *in vitro* proinflammatory effects.

One advantage of the current study is that acute ASP effects rather than chronic effects were evaluated. In previous studies, effects of chronic administration of ASP were evaluated in either C3KO or wild-type mice [27, 28], demonstrating effects on decreased insulin sensitivity, weight gain, and lipid/glucose metabolism. However, it is difficult to establish whether these effects are direct or indirect or a consequence of resistance induced by chronic stimulation. Similar concerns are an issue in knockout mice models where the proinflammatory effects [27, 29] may result from indirect disruptions in the immune system. Specifically, C3KO mice lack ASP but also lack an active complement system; C5L2KO mice lack ASP/C5L2 interaction but also lack C5a/C5L2 interaction which could disrupt the balance in C5a/C5aR-mediated effects.

The results shown in this study are not consequent to changes in body weight or food intake, which could have been confounding factors. We can therefore directly relate the changes observed to acute bolus ASP-mediated effects. The dose of ASP used was high physiological-pathological, as it theoretically could increase circulating ASP levels in obese mice by up to tenfold (from 400 nM to 4,2 *μ*M) and would mimic the high concentration of ASP generated following an acute immune activation of the complement system or seen in obese individuals. The most striking results obtained are the rapid, global increases in plasma inflammatory cytokines and the M1 polarization of tissue macrophages upon acute ASP treatment. We show additional *in vitro* evidence that supports a direct role for ASP in macrophage recruitment and M1 activation that is additive to classic cytokine effects. Notwithstanding the demonstrated proinflammatory ASP effects, acute ASP enhances plasma glucose excursion in DIO mice, with increased uptake into skeletal muscle and adipose tissue and reduced liver glucose uptake, and this effect is mediated in the presence of lower circulating insulin levels. While this could point towards increased insulin sensitivity, we also demonstrated that a single acute dose of ASP does not directly alter insulin sensitivity. We therefore speculate that the known *in vitro* activating effect of ASP on GLUT4 translocation in adipocyte and muscle cells [6] could explain the increase in muscle and adipose tissue glucose uptake, while passive concentration-dependent uptake in the liver, relying mostly on GLUT2 transporters, would indirectly be lowered. 

Our study, while demonstrating intriguing novel properties of ASP, is also consistent with previously published results. Chronic ASP supplementation decreased plasma NEFA, as in the present study [28]. An acute ASP bolus, on the other hand, has been shown to increase glucose excursion following a fat load [30], while C5L2^−/−^ (ASP receptor) deficient mice showed delayed glucose clearance [12]. Additionally, the potential chemoattractive role proposed for ASP [19, 20] has been demonstrated specifically in this study. He et al. also showed that high levels of ASP increased TNF-*α* and IL-6 liver expression [20]. We demonstrate here that this phenotype is also present in other tissues as well as in the circulation and extends to other major cytokines. By contrast, leptin levels were acutely decreased by ASP bolus. This effect could be potentially linked with the reduction of insulin secretion, a well-known stimulator of leptin release.

Inflammation and macrophage recruitment are inherent components of several biological systems related to tissue injury and infections and are beneficial in the short term. At the same time, the capacity of immune cells to exert their respective functions is modulated by their metabolic status. To label ASP acute effects as deleterious because of the proinflammatory capacities is likely an oversimplification of a potentially complex process. Certain cytokines exhibit both proinflammatory and glucose-sensitizing effects, while others have dual roles (that are either concentration dependent or time dependent) in immunity. Adiponectin is a well-known adipokine that improves insulin sensitivity but has been shown to exert both anti-inflammatory and proinflammatory effects, depending on the context [31]. IL-6 is another prime example of a cytokine with paradoxical effects; IL-6 increases glucose excursion through a short-term insulin-sensitizing process in spite of its role in inflammation [32, 33]. Long-term exposure to IL-6 may then, paradoxically, induce insulin resistance [34]. 

Humans with obesity, type 2 diabetes, and cardiovascular disease have been shown to exhibit higher chronic plasma ASP levels [2]. These morbidities are associated with macrophage infiltration and M1 polarization within several tissues. However, even in the absence of chronically increased ASP, adipose tissue acutely increases local ASP production postprandially [21, 22]. Thus, the response to an acute increase versus chronically elevated levels may differ and may depend on the concomitant diet (such as high-fat and high-sucrose diet). While it has been suggested that C3adesArg is simply an inactive immune by-product representative of complement activation, we clearly demonstrate in this study a direct role of ASP within these inflammatory processes. The relative contribution of acute ASP-stimulated inflammation in obesity-related diseases remains to be investigated. As with several signaling pathways in obesity, it could be that chronic substrate overload leads to excessive activation of the ASP signaling pathway, eliciting a deleterious response that could exacerbate the development of comorbidities through a low-grade proinflammatory phenotype transition [3]. 

## 5. Conclusion

The present study sheds new light on the dual role of ASP in energy metabolism and inflammation. ASP exhibits both substrate storage and proinflammatory properties that have been strongly associated with several pathologies. Effectively, neutralizing a hormone that can be both produced locally and acts locally represents a strong pharmaceutical challenge. A TNF-*α* inhibitor, for example, failed at altering insulin sensitivity in a human cohort [35]. Additional studies on C5L2, the only known receptor for ASP, are needed to better understand ASP-C5L2 signal transduction and to assess the potential of C5L2 as a pharmaceutical target in obesity and metabolic syndrome.

## Figures and Tables

**Table 1:** Real-time qPCR primers.

F4-80 forward	CTTTGGCTATGGGCTTCCAGTC
F4-80 reverse	GCAAGGAGGACAGAGTTTATCGTG
CD11c forward	CTGGATAGCCTTTCTTCTGCTG
CD11c reverse	GCACACTGTGTCCGAACTC
CD163 forward	GGGTCATTCAGAGGCACACTG
CD163 reverse	CTGGCTGTCCTGTCAAGGCT
IL-6 forward	GAGGATACCACTCCCAACAGACC
IL-6 reverse	AAGTGCATCATCGTTGTTCATACA
KC forward	TCTCCGTTACTTGGGGACAC
KC reverse	CCACACTCAAGAATGGTCGC
MCP-1 forward	ATTGGGATCATCTTGCTGGT
MCP-1 reverse	CCTGCTGTTCACAGTTGCC
MIP-1*α* forward	GTGGAATCTTCCGGCTGTAG
MIP-1*α* reverse	ACCATGACACTCTGCAACCA
PAI-1 forward	GCCAGGGTTGCACTAAACAT
PAI-1 reverse	GCCTCCTCATCCTGCCTAA
TNF-*α* forward	CATCTTCTCAAAATTCGAGTGACAA
TNF-*α* reverse	TGGGAGTAGACAAGGTACAACCC

Quantitative real-time PCR primer sequences used in evaluation of expression.

**Table 2:** Plasma lipids and cytokines.

Cytokine (unit)	Control (*n* = 8)	ASP (*n* = 7)
TG (mmol/L)	1.2 ± 0.2	1.1 ± 0.1
NEFA (mmol/L)	0.76 ± 0.07	0.53 ± 0.04*
IL-6 (pg/mL)	1.8 ± 0.9	22.8 ± 3.7***
IL-10 (pg/mL)	6.8 ± 2.3	18.6 ± 4.8^#^
G-CSF (pg/mL)	38.8 ± 7.7	70.2 ± 12.9^#^
GM-CSF (pg/mL)	14.3 ± 2.5	29.3 ± 5.9*
KC (pg/mL)	65.3 ± 12.2	403.4 ± 95.1**
MIP-1*α* (pg/mL)	3.2 ± 0.7	6.76 ± 1.2*
TNF-*α* (pg/mL)	39.6 ± 4.4	105.0 ± 24.1*
PAI-1 (ng/mL)	827 ± 167	1382 ± 84**
Leptin (*μ*g/mL)	16.1 ± 4.0	6.5 ± 2.2*

Plasma characteristics of mice, with/without ASP injection. Plasma lipids and cytokines were compared by *t*-tests. Results are expressed as mean ± sem where ^#^ *P* = 0.08, *P < 0.05, and **P < 0.01.

## Abbreviations

ASP: Acylation stimulating protein
C3aR: Complement C3a receptor
DIO: Diet-inducedobesity
GAPDH: Glyceraldehyde 3-phosphate dehydrogenase
G-CSF: Granulocyte colony-stimulating factor
GM-CSF: Granulocyte macrophage colony-stimulating factor
GTT: Glucose tolerance test
HF/HS: High-fat/high-sucrose
ITT: Insulin tolerance test
KC: Keratinocyte-derived chemokine
MCP-1: Monocyte chemoattractant protein-1
MIP-1*α*: Macrophage inflammatory protein-1*α*
NEFA: Nonesterified fatty acids
PAI-1: Plasminogen activator inhibitor-1
TG: Triglyceride.
